# Comparison of angiogenesis-related factor expression in primary tumor cultures under normal and hypoxic growth conditions

**DOI:** 10.1186/1475-2867-8-11

**Published:** 2008-07-10

**Authors:** Jamie M Heinzman, Stacey L Brower, Jason E Bush

**Affiliations:** 1 Research and Development, Precision Therapeutics, Inc., Pittsburgh, Pennsylvania, USA

## Abstract

**Background:**

A localized hypoxic environment occurs during tumor growth necessitating an angiogenic response or tumor necrosis results. Novel cancer treatment strategies take advantage of tumor-induced vascularisation by combining standard chemotherapeutic agents with angiogenesis-inhibiting agents. This has extended the progression-free interval and prolonged survival in patients with various types of cancer. We postulated that the expression levels of angiogenesis-related proteins from various primary tumor cultures would be greater under hypoxic conditions than under normoxia.

**Methods:**

Fifty cell sources, including both immortalized cell lines and primary carcinoma cells, were incubated under normoxic conditions for 48 hours. Then, cells were either transferred to a hypoxic environment (1% O_2_) or maintained at normoxic conditions for an additional 48 hours. Cell culture media from both conditions was collected and analyzed via an ELISA-based assay to determine expression levels of 11 angiogenesis-related factors: VEGF, PDGF-AA, PDGF-AA/BB, IL-8, bFGF/FGF-2, EGF, IP-10/CXCL10, Flt-3 ligand, TGF-β1, TGF-β2, and TGF-β3.

**Results:**

A linear correlation between normoxic and hypoxic growth conditions exists for expression levels of eight of eleven angiogenesis-related proteins tested including: VEGF, IL-8, PDGF-AA, PDGF-AA/BB, TGF-β1, TGF-β2, EGF, and IP-10. For VEGF, the target of current therapies, this correlation between hypoxia and higher cytokine levels was greater in primary breast and lung carcinoma cells than in ovarian carcinoma cells or tumor cell lines. Of interest, patient cell isolates differed in the precise pattern of elevated cytokines.

**Conclusion:**

As linear correlations exist between expression levels of angiogenic factors under normoxic and hypoxic conditions *in vitro*, we propose that explanted primary cells may be used to probe the *in vivo *hypoxic environment. Furthermore, differential expression levels for each sample across all proteins examined suggests it may be possible to build a predictor for angiogenesis-related anticancer agents, as each sample has a unique expression profile. Further studies should be performed to correlate *in vitro *protein expression levels of angiogenesis-related factors with *in vivo *patient response.

## Background

As a tumor grows, the existing blood supply becomes inefficient at supporting the tissue, and areas of the tumor become hypoxic. The hypoxic condition triggers the tumor to enhance the expression of angiogenic factors, triggering the formation of new blood vessels to support the growing tissue [1,2]. Angiogenesis is required for tumor survival as well as further growth, progression and metastasis [3]. In fact, high tumor vascular density is correlated with negative patient outcomes, including shorter progression-free interval and reduced overall survival [1,4,5].

One angiogenic factor whose expression is induced in response to a hypoxic environment is vascular endothelial growth factor (VEGF). VEGF belongs to the cystine-knot family of growth factors [4]. Four homologous polypeptides for VEGF exist, derived by alternative splicing of mRNA [2]. VEGF is secreted by cancer cells as well as supporting stromal cells, including fibroblasts, especially during conditions of hypoxia [1]. *In vitro *studies have shown that stromal cells cultured in hypoxic growth conditions secrete higher levels of critical angiogenesis-inducing factors than cells cultured in normoxic conditions [3]. High expression of VEGF is observed in many tumor types and is correlated with aggressive tumor growth and metastasis [5-7].

Regulation of VEGF is complex, occurring at both the transcription and translation stages of protein synthesis, with many ligand-receptor interactions [2,3,8]. Expression of VEGF is up-regulated by hypoxia inducible factor-1 (HIF-1), which binds to the VEGF promoter, increasing transcription of VEGF [9,10]. Once expressed, VEGF has the ability to bind to two endothelial cell-specific receptors, kinase domain receptor (KDR, VEGFR2) and fms-like tyrosine kinase (Flt-1, VEGFR1) to initiate angiogenesis among other survival signals [4,11]. While VEGF binds to Flt-1 with 50-fold higher affinity, KDR binding is more important for angiogenic responses [4]. Brogi et al. found hypoxia induced a 13-fold increase in the number of KDR receptors per endothelial cell *in vitro*, which may be the mechanism of action for the pronounced effect of hypoxia and VEGF *in vivo *[12]. In addition to simulating endothelial cell proliferation and migration, VEGF increases vasculature permeability, earning its other name as vascular permeability factor (VPF) [8,10,13]. This vascular leakage is critical for initiating angiogenesis as it allows proteins, such as matrix metalloproteases (MMPs), to be deposited in the extracellular fluid [9]. MMPs break down the extracellular matrix to enable endothelial cells to migrate and invade areas in close proximity to the tumor [9].

In addition to VEGF, a number of cytokines, chemokines, and growth factors are involved in angiogenesis. The eleven factors tested in this study, summarized in Table 1, were chosen because of their implication in altering vascular structure and the availability of Enzyme-Linked ImmunoSorbent Assays (ELISAs) for quantitative measurement. These angiogenesis-related factors fall into a number of general categories. Some work by mediating VEGF production, such as basic Fibroblast Growth Factor (bFGF/FGF-2) and Epidermal Growth Factor (EGF) [6,9,11,14]. Others work by modifying the extracellular environment of the tumor, including bFGF, Interleukin-8 (IL-8/CXCL8), and Platelet-derived Growth Factors-AA and -AA/BB (PDGFs) [6,15-17]. Induction of endothelial cell growth is accomplished by IL-8, Fms Related Tyrosine Kinase (Flt-3 Ligand), and PDGFs, while EGF and Transforming Growth Factors-β1, β2, and β3 (TGFs) are involved in tumor growth and proliferation [9,15,18-20]. Lastly, IP-10/CXCL10 inhibits tumor and endothelial cell growth and is inversely correlated with VEGF production [21].

**Table 1:** Description and role of angiogenesis-related factors.

**Angiogenesis-Related Factor**	**Role in Angiogenesis**
**Vascular Endothelial Growth Factor/Vascular Permeability Factor ** (VEGF/VPF)	Signalling protein for angiogenesis that works by binding, dimerizing, and phosphorylating external tyrosine kinase receptors. Can be induced by hypoxia through the release of Hypoxia Inducible Factor (HIF) [4,6,8,10,13].
**Basic Fibroblast Growth Factor ** (bFGF/FGF-2)	Stimulates production of basement membranes via formation of extracellular matrix. Aids in angiogenesis in tumors by mediating VEGF production [6,11,14].
**Interleukin-8 ** (IL-8/CXCL8)	A chemokine that regulates angiogenesis by promoting survival of endothelial cells, stimulating matrix metalloproteinases, and increasing endothelial permeability [15,16].
**Epidermal Growth Factor ** (EGF)	Factor commonly expressed in carcinomas involved in tumor growth, proliferation, and differentiation by stimulation of intrinsic protein-tyrosine kinase activity, resulting in DNA synthesis. Also, induces VEGF, IL-8, and bFGF release by tumor cells [9,19].
**Fms-related Tyrosine Kinase** (Flt-3 Ligand)	Cytokine that assists in proliferation and maturation of hematopoietic progenitor cells [20].
**Platelet-derived Growth Factors** (PDGF-AA, -AA/BB)	Mitogenic factors for fibroblasts, smooth muscle, and connective tissue that can be induced by VEGF and bFGF. Induce endothelial cell survival by recruiting stromal cells for VEGF production [9,17].
**Interferon-gamma-inducible Protein 10 ** (IP-10)	Inhibits tumor growth by regulating lymphocyte chemotaxis and inhibiting endothelial cell growth. Down-regulation correlated with poor prognosis. Reverse-correlated with VEGF [21].
**Transforming Growth Factors ** (TGF-β1,2,3)	Cytokines that control several biological processes including cell growth, proliferation, differentiation, and apoptosis. Pathological conditions such as cancer are can be linked to modifications of these growth factors [18].

VEGF production by tumor cells is of particular interest as this growth factor is being targeted by new anticancer agents. Specifically, Bevacizumab (Avastin^®^, Genentech) is a recombinant humanized monoclonal antibody, approved for the treatment of colorectal cancer and non-small cell lung cancer treatment by the FDA [5,7,22]. This drug binds VEGF with high specificity, neutralizing the growth factor and preventing the interaction of VEGF with its receptors. Therefore, proliferation of endothelial cells is inhibited and tumor progression is hindered [8,11].

Based on physiological *in vivo *conditions, it was hypothesized that cells grown in a hypoxic *in vitro *environment will express angiogenic factors, including VEGF, at higher levels than those grown under normoxic conditions. A secondary goal of this study was to determine whether primary tumors exhibit differential expression of angiogenic-related factors, a phenomenon which may be useful in predicting patient response to anti-angiogenic anticancer agents.

## Methods

### Primary cell cultures

Primary cell cultures were established using tumor specimens procured for research purposes from the following sources: National Disease Research Interchange (NDRI) (Philadelphia, PA), Cooperative Human Tissue Network (CHTN) (Philadelphia, PA), Forbes Regional Hospital (Monroeville, PA), Jameson Hospital (New Castle, PA), Saint Barnabas Medical Center (Livingston, NJ), Hamot Medical Center (Erie, PA), and Windber Research Institute (Windber, PA). Upon receipt, all specimens were minced to a fine consistency with Cincinnati Surgical #10 or #11 scalpels (PGC Scientifics, Frederick, MD), followed by antibiotic washes, as necessary. In order to establish primary cultures, the specimens were typically divided into 25 cm^2 ^and/or 75 cm^2 ^Cellstar^® ^sterile tissue culture flasks with filtered caps (PGC Scientifics, Frederick, MD), depending on the desired seeding density. Cell culture media were tumor type specific: breast tumors were cultured in Mammary Epithelial Growth Media (MEGM; Lonza Bio Science Walkersville, Walkersville, MD), ovarian tumors were cultured in McCoy's 5A growth media (Mediatech, Herndon, VA), lung tumors were cultured in Bronchial Epithelial Growth Media (BEGM; Lonza Bio Science Walkersville), and colon tumors were cultured in RPMI 1640 growth media (Mediatech). The amount of Fetal Bovine Serum (FBS; HyClone, Logan, UT) present in the media was also tumor-type specific, as was the presence of PureCol™ collagen (Inamed Biomaterials, Fremont, CA) on the culture surface. Antibiotic washes and antibiotic media were formulated with Penicillin-Streptomycin Solution (Mediatech), Gibco Gentamicin Reagent Solution (Invitrogen Corporation, Grand Island, NY), Fungisone (Invitrogen), Cipro^® ^I.V. (ciprofloxacin) (Oncology Therapeutics Network, South San Francisco, CA), and Nystatin (Sigma-Aldrich, St. Louis, MO). Other reagents include Trypsin EDTA (0.25%) and Hanks Buffered Saline Solution with and without Calcium and Magnesium (HBSS) (Mediatech).

All cultures were initially established in humidified incubators at 37°C with 5% CO_2 _for 5 to 28 days. When a confluency of at least 30 percent was attained, cells were trypsinized, counted, and plated as described below.

### Established cell lines

Three human tumor-derived immortalized cell lines were also tested in this study: SK-OV-3, ovarian adenocarcinoma; MDA-MB-231, mammary adenocarcinoma; and A549, lung carcinoma (American Type Culture Collection, Manassas, VA). These cell lines were seeded at 50,000 cells per 5 ml in T25 flasks and allowed to grow for one week to approximately 90% confluency. At that time, the cells were trypsinized, counted, and plated as described below.

### Testing conditions

After the initial culture period, a total of fifty samples (45 primary cultures and 5 cell line samples) were trypsinized, counted, and suspended in culture media to a concentration of 40,000 cells/ml. SK-OV-3 was tested on three separate occasions to ensure consistency of results. Each of the samples was plated at 20,000 cells/well into one well of two separate Greiner 24-well culture plates (CLP Molecular Biology, San Diego, CA). Both plates were maintained under normoxic conditions (5% CO_2 _and 21% O_2_) for 48 hours to allow for cell adherence and equilibration. After 48 hours, one plate remained in normoxic conditions while the other plate was transferred to a NAPCO Series 8000WJ Water Jacketed CO_2 _Incubator (ThermoFisher Scientific, Waltham, MA) where hypoxic conditions were established. Nitrogen gas was injected to purge the incubator of oxygen resulting in a final O_2 _concentration of 1% while the CO_2 _concentration was maintained at 5%, as described by Mukherjee et al. [3]. Plates were incubated for an additional 48 hours. At the end of the incubation period, the confluency for each sample was recorded and the supernatant was collected and stored at -80°C. Confluency is the percentage of substrate with adherent cell growth, determined subjectively by a trained technician.

### ELISA

Collected supernatants were sent to Millipore Corporation (Temecula, CA) for protein evaluation via the Beadlyte^® ^CytokineProfiler™ Testing Service, an ELISA-based assay. Evaluated angiogenesis-related cytokines and growth factors included: VEGF, PDGF-AA, PDGF-AA/BB, IL-8, bFGF, EGF, IP-10, Flt-3 ligand, TGF-β1, TGF-β2, and TGF-β3. Additionally, RANTES (Regulated upon Activation, Normal T-cell Expressed, and Secreted), an analyte not related to angiogenesis, was tested as a negative control for a subset of samples [23]. For each analyte, two replicates were performed using 40 μl of supernatant per replicate.

### Statistical analysis

For each analyte, protein expression levels in the normoxic and hypoxic conditions of all samples were combined into a scatter plot. Then, a linear regression of the curve fit for protein concentration under the hypoxic versus normoxic condition was generated for each analyte tested. For all linear regressions, y = mx+b, y is the concentration produced in the hypoxic environment and x is the concentration produced in the normoxic condition. From this regression, the slope, intercept, and correlation of determination (r^2^) were calculated. The strength of each linear relationship was determined by the r^2 ^value of the linear regression, with r^2 ^values greater than 0.8 considered strong relationships, and r^2 ^values between 0.6 and 0.8 considered moderate relationships. The same parameters were used to assess VEGF expression levels by tumor type. Lastly, comparisons were generated between the eleven angiogenesis-related factors studied for every cell source. The differences between the protein expression levels under the hypoxic condition versus the normoxic condition were calculated. This value was standardized on a scale of zero to one, with zero set equal to the lowest value observed and one set equal to the highest value observed. These values were graphed as a heat map for all samples across all factors. Additionally, Pearson correlation coefficients were calculated for each factor in relation to VEGF expression using the standardized differences between the hypoxic and normoxic expression levels.

## Results

### Patient specimens and cell lines

**Figure 1:**
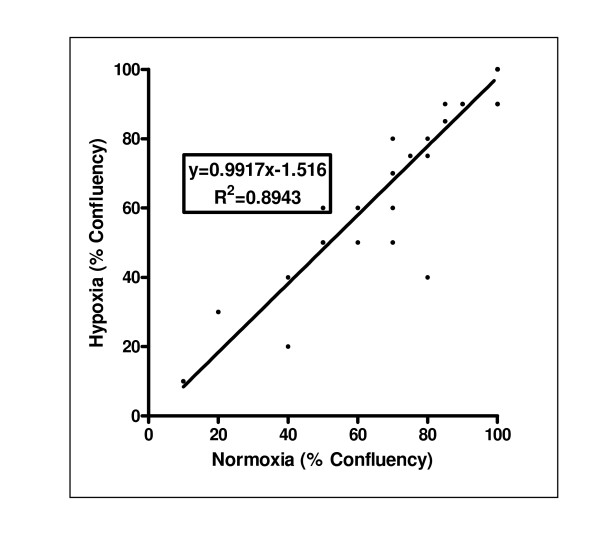
**Culture growth is comparable under normoxic and hypoxic conditions**. A linear regression of the normoxic versus hypoxic percent confluency of each of the 50 samples in the study shows the confluencies to be similar within a given sample. Many samples reached 100% confluency in both conditions, so less than 50 points appear on the graph.

The study included fifty distinct cell populations. Forty-five primary tumor specimens were designated based on final pathology and site of tumor origin including: 10 breast, 15 lung, 13 ovary, 3 colon, 3 central nervous system (CNS), and 1 unknown primary. Additionally, five cell line samples were tested including: A549, one sample; MDA-MB-231, one sample; and SK-OV-3, three samples. All samples were evaluated under both normoxic and hypoxic environments in parallel. A strong linear relationship for the confluency of the normoxic versus hypoxic condition existed across all samples, with a linear regression of y = 0.9917x-1.516 (r^2 ^= 0.8943; Figure 1).

### Hypoxia-induced expression of angiogenesis-related factors

Moderate to strong linear relationships of the protein expression levels between hypoxic and normoxic conditions were observed in eight of the eleven angiogenesis-related factors analyzed (Table 2). The strongest linear relationships (r^2 ^> 0.95) are evident for IL-8, with hypoxic expression levels generally higher than normoxic (m = 0.9627, b = 569.1), and PDGF-AA, with lower levels in hypoxia (m = 0.8322, b = -1.859). Strong correlations (r^2 ^> 0.80) existed for a number of growth factors (all expressing similar levels under hypoxia and normoxia conditions), including: EGF (m = 0.9497, b = -70); TGF-β2 (m = 0.9632, b = 22.65); and PDGF-AA/BB (m = 1.015, b = 3.74). One anti-angiogenic factor, IP-10, also had a strong linear correlation, with hypoxic expression levels lower than normoxic (m = 0.8778, b = -27.55). Moderate correlations (r^2 ^> 0.60) were observed for VEGF, with higher levels in hypoxia (m = 1.174, b = 552.2), and TGF-β1 (m = 0.6186, b = 194.7), with lower levels in hypoxia than normoxia. Linear correlations did not exist for bFGF or TGF-β3 (r^2 ^< 0.25). Data for Flt-3 ligand was not evaluable, as only six of 50 samples had evaluable results. RANTES, tested in six samples, indicated similar expression levels for both conditions (y = 1.0411x+0.0807 and r^2 ^= 0.9924) suggesting that the changes noted in the other cytokines were due to hypoxia.

**Table 2:** Linear correlations between normoxic and hypoxic growth conditions of angiogenesis-related factors.

**Analyte**	**n**	**Slope ** **(m)**	**95% CI Slope (m)**	**y-intercept ** **(b)**	**95% CI y-** **intercept**	**r**^2^
**VEGF**	50	1.174	0.9049 to 1.443	552.2	98.99 to 1005	0.6163
**bFGF**	27	0.0813	-0.06828 to 0.2309	82.38	50.21 to 114.5	0.0478
**IL-8**	33	0.9627	0.9076 to 1.018	569.1	2.366 to 1136	0.9761
**EGF**	22	0.9497	0.8266 to 1.073	-70	-357.1 to 217.1	0.9283
**PDGF-AA**	48	0.8322	0.7925 to 0.8720	-1.859	-20.92 to 17.21	0.9748
**PDGF-AA/BB**	21	1.015	0.8348 to 1.196	3.74	-102.7 to 110.1	0.8793
**IP-10**	35	0.8778	0.7738 to 0.9817	-27.55	-292.3 to 237.1	0.8995
**TGF-β1**	45	0.6186	0.4914 to 0.7458	194.7	93.33 to 296.1	0.6913
**TGF-β2**	47	0.9632	0.8808 to 1.045	22.65	-226.8 to 272.1	0.9251
**TGF-β3**	27	0.2433	-0.1484 to 0.6350	23.36	10.64 to 36.07	0.0615

### Hypoxia-induced expression of VEGF is tissue-type dependent

**Figure 2:**
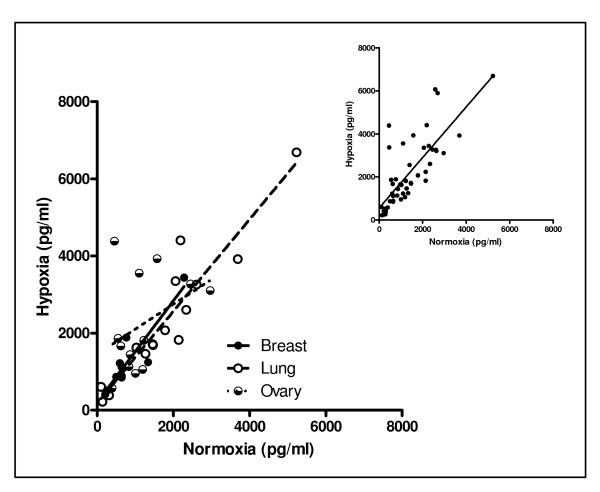
**Linear correlations between normoxic and hypoxic conditions exist for VEGF and group by tumor type**. Fifty cell sources (45 primary tumor cultures and 5 immortalized cell lines) were evaluated for VEGF expression measured by ELISA-based assay. Linear correlations exist between cells grown in normoxic and hypoxic conditions (Table 2). The larger graph divides the specimens by tumor type, while the inset combines all data sets.

For VEGF, 46 of 50 samples exhibited higher expression levels in the hypoxic condition than in the normoxic condition. Since VEGF is the angiogenesis-related factor specifically implicated in the mechanism of action of bevacizumab, this data was further analyzed by tissue type (Figure 2, Table 3). Overall, the combined results of all cell sources analyzed had a moderate correlation (r^2 ^> 0.60). Breast, lung, and ovarian tumor types had sufficient sample sizes to sub-analyze by tumor type. While strong linear correlations were observed for breast and lung samples (r^2 ^> 0.80), a linear correlation between hypoxic and normoxic expression of VEGF in ovarian samples did not exist (r^2 ^< 0.25). Linear correlations were not available for CNS, colon and unknown primary tumors or for the cell lines, as samples sizes were too low to assess linearity.

**Table 3:** Linear correlations of VEGF between normoxic and hypoxic conditions.

**VEGF Results**	**n**	**Slope ** **(m)**	**95% CI Slope**	**y-intercept** **(b)**	**95% CI y-** **intercept**	**r**^2^
**Breast**	10	1.316	0.8360 to 1.795	206.1	-262.5 to 674.7	0.8334
**Lung**	15	1.193	0.9280 to 1.458	178.1	-422.0 to 778.3	0.8793
**Ovary**	13	0.6432	-0.3679 to 1.654	1458	58.40 to 2858	0.1513
**All Samples**	50	1.174	0.9049 to 1.443	552.2	98.99 to 1005	0.6163

### Differential expression of angiogenesis-related factors across patient samples

**Figure 3:**
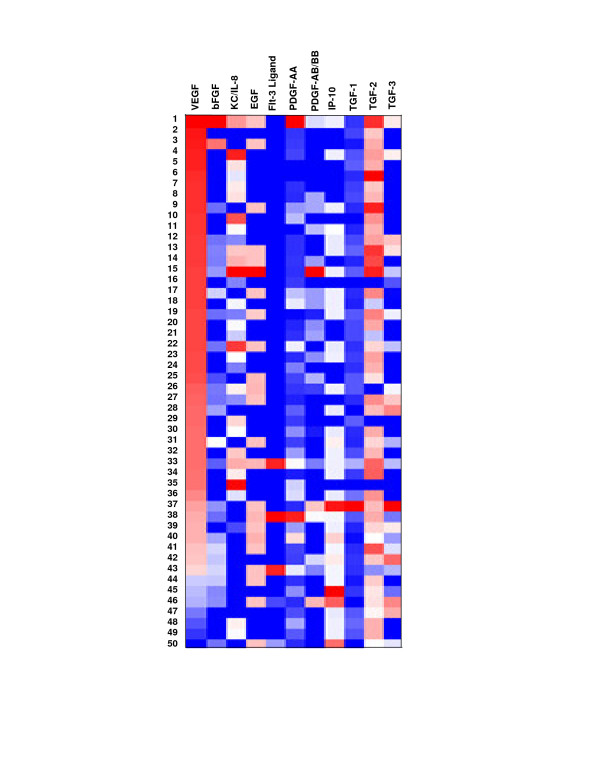
**Differential expression of angiogenesis-related factors is evident across samples**. Differential levels of expression are evident across all patients for the angiogenesis-related factors tested. Bright red indicates lowest expression level difference, bright blue indicates highest expression level difference, and white indicates no data. Correlation coefficients indicate the differences in VEGF are not correlated to differences in expression of the other angiogenesis-related proteins.

A heat-map of the differences between hypoxic and normoxic expression indicates expression levels of angiogenesis-related factors differed both within and between patients (Figure 3). This data was specifically sorted by VEGF expression from lowest to highest difference for a visual representation of the heterogeneous expression levels. Pearson correlation coefficients were also calculated for all nine angiogenesis-related factors with evaluable data in relationship to VEGF (data not shown). The Pearson correlation coefficients were all less than 0.5, indicating differences in the other angiogenesis-related factors are not correlated to differences in VEGF expression. Together, these data reinforce the idea that differential angiogenesis-related protein expression levels exist for each sample.

## Discussion

This study addressed a number of topics related to the expression of angiogenesis-related factors in normoxic versus hypoxic environments. Specifically, (1) linear correlations exist for a number of angiogenesis-related factors, (2) linear correlations for VEGF exist and group by tumor type, and (3) primary expression levels vary between samples and across factors.

Linear correlations between protein expression in normoxic and hypoxic environments exist for eight of the eleven angiogenesis-related factors tested in this study (Table 2). Hypoxic expression levels were generally higher than normoxic for IL-8 (r^2 ^> 0.95) and VEGF (r^2 ^> 0.60), though only modestly. Both of these factors are expressed to induce vascular growth due to hypoxia *in vivo*, and appear to do the same *in vitro*. The degree of difference was surprising, as both IL-8 and VEGF have been reported to be up-regulated in response to hypoxic conditions. IL-8 regulates angiogenesis by promoting survival of endothelial cells, stimulating matrix metalloproteinases, and increasing endothelial permeability [15,16]. VEGF is a major signalling protein for angiogenesis secreted in higher levels when cells experience hypoxia [4]. IP-10, an anti-angiogenic factor, had lower expression levels in the hypoxic condition than in the normoxic condition (r^2 ^> 0.80). This was expected, as this protein inhibits tumor growth by regulating lymphocyte chemotaxis and inhibiting endothelial growth [21].

Trends in the expression levels of other growth factors were variable. Lower expression levels were observed in the hypoxic condition for PDGF-AA (r^2 ^> 0.95) and similar levels were observed for PDGF-AA/BB (r^2 ^> 0.80). These results are not surprising as platelet populations are minimal in culture. These cells are non-adherent to flask surfaces and are rinsed away during routine media changes. Different results were observed for each of the transforming growth factors, likely related to the specific role each plays in cancer pathogenesis [18]. Lower expression levels were observed in the hypoxic condition for TGF-β1, while similar expression levels were observed in both conditions for TGF-β2 (r^2 ^> 0.80) and no correlation existed for TGF-β3 (r^2 ^< 0.25). Similar expression levels were observed in both conditions for EGF, which may be due to the fact that EGF induces VEGF, IL-8, and bFGF release by tumor cells, and is transformed in the process [9]. A correlation did not exist for bFGF, which mediates VEGF production and induces extracellular matrix formation. Another *in vitro *study showed bFGF was unaffected by hypoxia in cell lines [3]. In all, the correlations between the conditions *in vitro *suggest the expression levels may be linked to *in vivo *expression of each angiogenesis-related factor, whether measured in normoxic or hypoxic conditions.

The combined results of all cell sources analyzed for VEGF showed a moderate correlation between normoxic and hypoxic expression levels. Stronger linear correlations were observed for breast and lung samples specifically. Breast and lung samples are cultured in unique culture media as compared to ovarian, CNS, and colon samples. Primary breast tumors are cultured in Mammary Epithelial Growth Media (MEGM), while lung tumors are cultured in Bronchial Epithelial Growth Media (BEGM). These media require addition of SingleQuots^® ^to basal media that include EGF. Significantly, EGF induces VEGF, IL-8, and bFGF release by tumor cells [9]. While this SingleQuots^® ^may have contributed to the VEGF production in these tumor types, the other analytes (IL-8 and bFGF) induced by EGF did not correlate by tumor type (data not shown). Therefore, culture media is probably not responsible for the differential expression levels of the ten evaluable angiogenesis-related proteins and a unique fingerprint for each sample. In general, these data suggest *in vitro *expression levels of VEGF can be measured in either a normoxic or a hypoxic condition, since a linear correlation exists between expression levels in both conditions.

Differential protein expression levels existed for each factor tested in this study, as is evident in Figure 3. *In vitro *studies show differential degrees of primary tumor response to chemotherapy agents. These response rates correlate with progression-free interval in ovarian cancer patients, which indicates *in vitro *tests performed on primary cultures may be used to enhance the probability of choosing the best treatment regimen for the patient [24]. Similarly, differential protein expression levels were observed across patients in this study for each of the factors. This suggests it may be possible to build a predictor for angiogenesis-related anticancer agents using an array of protein expression levels observed *in vitro*.

There are limits to the application of these *in vitro *results to the *in vivo *condition. The tumor microenvironment *in vivo *is unique both in its three-dimensional structure and the chemical environment [25]. This affects cellular behaviour, including response to chemotherapeutic agents. Some researchers have successfully developed culture systems that replicate this three-dimensional interaction of cells [26]. This study, however, employed a monolayer culture system specifically designed to enrich the population of malignant epithelial cells [27]. While toxicity, delivery, metabolism, and clearance affect patient response to therapeutics *in vivo*, *in vitro *studies are commonly used in initial testing of novel treatments and have clinical potential when applied [28].

Although extreme hypoxic conditions may compromise the health of the cells and lead to cell death, similar confluencies between the normoxic and hypoxic condition at the conclusion of testing suggest that the 48 hour incubation prior to testing was sufficient for cell adherence and equilibration (Figure 1). To support this observation, Pilch et al. found that hypoxia did not cause cell death/decreased confluency, as dead cells were not observed in supernatant post-hypoxia [1]. Furthermore, the hypoxic oxygen concentration used in the study *in vitro *is similar to that reported by Hockel and Vaupel, 2001, in the core of solid tumors *in vivo *[29].

Also, in addition to the 11 angiogenesis-related analytes chosen for testing, a negative control unrelated to angiogenesis was also assessed. A chemotactic cytokine, RANTES, is responsible for recruiting leukocytes and activating natural killer cells [23]. This cytokine was not expected to vary in a normoxic versus hypoxic environment, and we found similar expression levels of RANTES for both conditions validating our technical approach.

Multiple techniques are available to assess VEGF expression. Some laboratories employ immunohistochemical (IHC) analysis to determine VEGF receptor levels [5], usually for diagnostic and prognostic purposes. However, this study employed the Beadlyte^® ^CytokineProfiler™ Testing Service for two reasons. First, this service provides quantitative analysis of the expression levels of the angiogenesis-related factors, including VEGF. Second, testing was performed on malignant epithelial cell cultures, rather than tissue sections. Intact tissue sections that contain tumor cells as well as support tissue and vasculature are generally stained using IHC. VEGF receptors on endothelial cells and monocytes fluoresce. The described culture process selects specifically for malignant epithelial cells [27]. Endothelial cells are selected against by culture conditions, as the media employed do not promote the growth of these cells; monocytes are non-adherent, so are rinsed away in routine media changes [27]. Neither of these cell types is present in the described samples so IHC of the VEGF receptors was not possible.

VEGF production was of most interest to this study due to its role in the mechanism of action of bevacizumab. The testing conditions were optimized to ensure that VEGF production was measurable, so VEGF results were available for all samples tested. Table 3 includes the summary of all data in the "All Samples" field, a total of 50 samples. Results for the other ten analytes had detection levels out of range of the standard curve for at least two samples, if not more. As a result, the sample size for most of these angiogenesis-related factors was less than 50 (Table 2). However, nine of these ten factors had at least 20 samples available for analysis, and were considered evaluable in the study.

As with any anticancer therapeutic agent, there is clinical ambiguity regarding individual patient response. Some agents directly target VEGF, such as bevacizumab, a humanized monoclonal antibody, while others indirectly target receptors and downstream regulators, such as sunitinib and rituximab [8]. While the regulation and metabolism are unique *in vivo*, the protein expression levels produced by individual patient cells may provide information on how each patient will respond clinically to a given anticancer agent. The heterogeneity of protein expression demonstrated in this study may provide information to enable the prediction of the efficacy of anti-angiogenic factors. Further studies correlating the *in vitro *expression levels with patient outcome are warranted.

## Conclusion

Linear correlations exist between expression levels of angiogenesis-related factors under normoxic and hypoxic conditions. This suggests the behaviour of primary cells derived from patient tumors grown under *in vitro *normoxic conditions may provide a correlation to the *in vivo *hypoxic environment. Differential expression for each sample across all factors suggests predictive value for angiogenesis-related anti-cancer agents, using not only VEGF, but an array of angiogenesis-related proteins. These data suggest further studies should be considered to correlate *in vitro *expression of these proteins with *in vivo *patient response to anti-angiogenesis therapeutics.

## Competing interests

Drs. Brower and Bush, and Ms. Heinzman are employees of Precision Therapeutics, Inc. and have declared a financial interest in the company, which supported this work.

## Authors' contributions

JH performed the cell culture studies and drafted the manuscript, SB participated in the design of the study and review of the manuscript, and JB coordinated the manuscript and assisted in statistical analysis.
